# Anthropometric Measurements to Predict Metabolic Syndrome in Thai Women With Polycystic Ovary Syndrome: A Retrospective Study

**DOI:** 10.1111/jog.70255

**Published:** 2026-03-19

**Authors:** Marisa Yokyongsakul, Pavarit Humart, Kitirat Techatraisak, Prasong Tanmahasamut, Manee Rattanachaiyanont, Suchada Indhavivadhana, Thanyarat Wongwananuruk, Panicha Chantrapanichkul, Matus Phunyammalee, Pornpimol Madeesukstit

**Affiliations:** ^1^ Department of Obstetrics and Gynecology Faculty of Medicine Siriraj Hospital, Mahidol University Bangkok Thailand; ^2^ Gynecologic Endocrinology Unit, Department of Obstetrics and Gynecology Faculty of Medicine Siriraj Hospital, Mahidol University Bangkok Thailand

**Keywords:** abdominal volume index, anthropometry, body roundness index, metabolic syndrome, polycystic ovary syndrome

## Abstract

**Aim:**

To compare the diagnostic accuracy of seven anthropometric indices for predicting metabolic syndrome (MetS) in Thai women with polycystic ovary syndrome (PCOS).

**Methods:**

Electronic medical records of 1492 women aged 15–45 years who attended Siriraj Hospital during 2015–2024 were retrospectively analyzed. MetS was diagnosed using the International Diabetes Federation 2006 criteria. The indices assessed were a body shape index (ABSI), abdominal volume index (AVI), body adiposity index (BAI), body mass index (BMI), body roundness index (BRI), waist‐to‐hip ratio (WHR), and waist‐to‐height ratio (WHtR). Discrimination was evaluated by receiver operating characteristic (ROC) analysis with area under the curve (AUC) and 95% CI. Optimal cut‐offs were identified using the Youden index.

**Results:**

Metabolic syndrome prevalence was 20.8%. AVI, BMI, BRI, and WHtR showed comparable discrimination (AUC 0.84–0.85) and outperformed BAI (AUC 0.79), WHR (AUC 0.78), and ABSI (AUC 0.52). A composite of AVI, BRI, and WHtR did not improve discrimination. A BMI threshold of 25.78 kg/m^2^ provided the best overall accuracy (69.6%). A WHtR threshold of 0.53 offered similar clinical utility.

**Conclusions:**

BMI and waist‐centered indices demonstrate comparable performance for predicting MetS in Thai women with PCOS. BMI provides the highest overall accuracy and, together with WHtR, can serve as practical first‐line triage tools.

**Abbreviations**
ABSI: a body shape index
AMH: anti‐Müllerian hormone
AVI: abdominal volume index
BAI: body adiposity index
BMI: body mass index
BRI: body roundness index
CT: computed tomography
CVD: cardiovascular disease
HC: hip circumference
HDL‐C: high‐density lipoprotein cholesterol
Ht: height
IDF: International Diabetes Federation
LDL‐C: low‐density lipoprotein cholesterol
MetS: metabolic syndrome
MRI: magnetic resonance imaging
OD: ovarian dysfunction
PCOM: polycystic ovarian morphology
PCOS: polycystic ovary syndrome
T2DM: type 2 diabetes mellitus
VAI: visceral adiposity index
VAT: visceral adipose tissue
WC: waist circumference
WHR: waist‐to‐hip ratio
WHtR: waist‐to‐height ratio

## 1 Introduction

Polycystic ovary syndrome (PCOS) is the most common endocrine disorder among reproductive‐aged women, affecting approximately 5%–15% of this population worldwide [1, 2, 3]. While it is traditionally recognized for its reproductive manifestations, PCOS is now understood as a chronic metabolic condition. It is strongly associated with a cluster of cardiometabolic abnormalities including insulin resistance, dyslipidemia, hypertension, and visceral adiposity that collectively define metabolic syndrome (MetS). Epidemiological evidence indicates that women with PCOS have a 2–11‐fold higher risk of developing MetS compared with eumenorrheic women [4, 5]. In hospital‐based cohorts in Thailand, the prevalence of MetS among patients with PCOS has been reported to range from 20% to 30% [6, 7, 8].

Importantly, MetS is not confined to PCOS and represents a growing public health concern among reproductive‐aged women more broadly. MetS affects an estimated one‐quarter of adults worldwide and contributes to the early trajectory of type 2 diabetes mellitus (T2DM) and cardiovascular disease (CVD) risk across the life course [9]. Approximately 60%–70% of individuals with PCOS are classified as overweight or obese [10, 11, 12, 13, 14]. Obesity, particularly visceral adiposity, plays a central role in the pathophysiology of MetS [15]. Consequently, anthropometric indicators that reflect body shape and fat distribution have garnered increasing clinical significance. Advanced imaging modalities, such as computed tomography (CT) and magnetic resonance imaging (MRI), have consistently demonstrated that waist circumference (WC) and related indices are more strongly correlated with visceral adipose tissue (VAT) than body mass index (BMI) [16]. These findings underscore the practical value of WC as a surrogate marker for visceral fat, particularly in clinical settings where direct imaging assessments are not readily available.

Increasing evidence indicates that different adipose tissue depots exert distinct metabolic effects. Visceral fat, for example, is metabolically active and contributes to pathophysiological processes by promoting lipolysis and ectopic fat accumulation, thereby exacerbating insulin resistance and other metabolic abnormalities. In contrast, gluteofemoral fat may confer metabolic protection, potentially due to its lower lipolytic activity and its role as a long‐term fat storage site [17]. Among various anthropometric indicators, visceral obesity remains one of the most robust predictors of MetS and cardiovascular risk. However, WC includes both subcutaneous and visceral fat components, limiting its specificity in distinguishing between these two anatomically and functionally distinct compartments. This limitation poses a significant challenge in accurately estimating VAT, which has a stronger association with adverse metabolic outcomes than subcutaneous adiposity [18]. To overcome this issue, the International Diabetes Federation (IDF) advocates imaging‐based methods such as CT, MRI, and dual‐energy X‐ray absorptiometry as gold standards for VAT assessment [19]. Despite their high accuracy, these techniques are constrained by their high costs, limited accessibility, and concerns related to radiation exposure, making them unsuitable for widespread use in routine clinical practice. As such, the identification of reliable, low‐cost, and noninvasive surrogate markers for VAT remains a critical need.

In response, several novel anthropometric indices have been developed to more accurately reflect fat distribution and metabolic risk. For example, the abdominal volume index (AVI) integrates WC and hip circumference (HC) to estimate abdominal fat volume, while the body adiposity index (BAI) estimates the percentage of total body fat using HC and height. Other indices, such as a body shape index (ABSI), body roundness index (BRI), waist‐to‐hip ratio (WHR), and waist‐to‐height ratio (WHtR), have been proposed to quantify fat distribution and overall body morphology. These indices have demonstrated significant associations with insulin resistance and dyslipidemia, particularly among women with PCOS [20, 21, 22, 23].

The IDF 2006 definition of MetS places central obesity at the core of the MetS. It requires ethnicity‐specific WC cut‐offs ≥ 80 cm for Southeast Asian women along with any two additional metabolic abnormalities [19]. Patients diagnosed with MetS face a significantly increased risk of developing T2DM, CVD, and certain types of cancer [24, 25]. Given these elevated health risks, several scientific organizations recommend periodic screening of patients with PCOS, a population known to be at increased risk for metabolic complications, to facilitate early detection of MetS [5, 26, 27].

Despite these recommendations, standard screening methods for MetS (e.g., laboratory testing and blood pressure assessment combined with diagnostic criteria) can be difficult to implement at scale because they are time‐consuming and may be constrained by staffing, patient throughput, and resource availability in routine outpatient settings. In Thailand, where many reproductive‐aged women with PCOS are managed in high‐volume gynecologic endocrinology clinics, there is a practical need for simple, rapid, and low cost screening approaches that can be applied at the point of care to identify those who warrant confirmatory metabolic evaluation.

Furthermore, reliance on single measures such as WC alone may be insufficient because body fat distribution and cardiometabolic risk profiles can vary across ethnicities and populations, potentially affecting the performance of anthropometric cut‐offs and indices. Therefore, identifying anthropometric indicators that are valid and clinically useful specifically in Thai women with PCOS is important to support tailored screening strategies tools that can be integrated into routine clinical encounters, facilitate early risk stratification, and guide timely referral for comprehensive cardiometabolic assessment.

Among these anthropometric indicators, beyond WHR and WHtR, newer indices such as ABSI, AVI, BAI, and BRI have emerged as potential tools for MetS risk prediction. However, the individual and combined diagnostic performance of these indices for predicting MetS among Thai women with PCOS remains unclear. Accordingly, the present study aims to compare the discriminative ability and clinical utility of these anthropometric indices to predict MetS, as defined by the IDF 2006 criteria.

## 2 Materials and Methods

### 2.1 Study Design and Setting

A retrospective cross‐sectional study was performed using data from the Siriraj PCOS project, which is the registry of PCOS women at the Gynecologic Endocrinology Unit, Faculty of Medicine Siriraj Hospital, Bangkok, Thailand. Siriraj Hospital is a tertiary‐care university hospital. The study period spanned January 1, 2015 to December 31, 2024. The approval for data analysis was granted by the Siriraj Hospital Institutional Review Board (COA. no. Si 927/2024) and the requirement for informed written consent was waived.

### 2.2 Participants

#### 2.2.1 Inclusion Criteria

Women aged 15–45 years with a new diagnosis of PCOS based on the Rotterdam Criteria 2003 were included. The diagnosis required the presence of at least two of the following features after exclusion of related disorders: (1) ovarian dysfunction (oligo‐/anovulation), (2) clinical and/or biochemical hyperandrogenism, and (3) polycystic ovarian morphology on transvaginal ultrasonography [28]. Eligible participants were required to be treatment‐naïve for PCOS and/or MetS, or to have discontinued any medication that affects blood pressure, sex hormones, glucose, or lipid metabolism for at least 3 months before enrollment. The upper age limit of 45 years was chosen to focus on reproductive‐aged women and to reduce confounding from perimenopausal hormonal changes and age‐related metabolic risks.

#### 2.2.2 Exclusion Criteria

Individuals were excluded if they were pregnant or lactating at the time of presentation, had a history of bariatric surgery, or were diagnosed with chronic hepatic disease or chronic kidney disease. Baseline anthropometric data or fasting laboratory results obtained more than 30 days after PCOS diagnosis were excluded.

#### 2.2.3 Sample Size

The sample size was calculated based on the estimated sensitivity of WHtR and BMI to detect MetS using the Buderer method [29], with an expected sensitivity of 0.88 [30], a MetS prevalence of 0.21 [6], an absolute precision of 0.05, and a 99% confidence level. The minimum required analyzable sample was 1338 participants. To account for potential incompleteness in medical charts in this retrospective review, the target sample size was inflated by 15% to 1538 records. Records with missing anthropometric or fasting laboratory data required for MetS classification were excluded (complete‐case analysis) and no imputation was performed.

### 2.3 Definition and Measurements

#### 2.3.1 Definition of MetS

MetS was diagnosed using the IDF 2006 criteria: mandatory central obesity (WC ≥ 80 cm for Southeast Asian women) plus any two of (1) fasting triglycerides ≥ 1.7 mmol/L or treatment; (2) HDL‐C < 1.29 mmol/L or treatment; (3) blood pressure ≥ 130/85 mmHg or antihypertensive therapy; and (4) fasting plasma glucose ≥ 100 mg/dL or previously diagnosed diabetes [19].

#### 2.3.2 Derived Anthropometric Indices

ABSI, AVI, BAI, BMI, BRI, WHR, and WHtR were calculated from WC, HC, weight, and height using standard formulas (Table 1).

**TABLE 1:** Index formulas.

Index	Formula
ABSI	WC (cm)/[BMI (kg/m^2^)^2/3^ × Ht (cm)^1/2^]
AVI	{2 × WC (cm)^2^ + 0.7 × [WC (cm) − HC (cm)]^2^}/1000
BAI	[HC (cm)/Ht (m)^1.5^] − 18
BMI	Wt (kg)/Ht (m)^2^
BRI	364.2–365.5 × √ [1 − (WC (cm)/2*π*)^2^/0.5 × Ht (cm)^2^]
WHR	WC (cm)/HC (cm)
WHtR	WC (cm)/Ht (cm)

Abbreviations: ABSI, a body shape index; AVI, abdominal volume index; BAI, body adiposity index; BMI, body mass index; BRI, body roundness index; cm, centimeter; HC, hip circumference; Ht, height; kg, kilogram; m, meter; WC, waist circumference; WHR, waist‐to‐hip; WHtR, waist‐to‐height ratio; Wt, weight.

#### 2.3.3 Anthropometric and Blood Pressure Measurements

The height of participants was measured using a stadiometer (TANITA W830, TANITA Corporation, Tokyo, Japan) while standing in an upright position with bare feet and the back against the stadiometer. Body weight was measured using a digital scale (TANITA W830, TANITA Corporation, Tokyo, Japan). Blood pressure was measured using an automated digital device (A&D Medical TM‐2657P, A&D Company, Saitama, Japan) after the participant had been seated at rest for at least 5 min. WC was measured at the midpoint between the lowest rib and the iliac crest at the end of a normal expiration. HC was measured at the level of the greater trochanters. A nonelastic measuring tape (SECA 201, SECA, Hamburg, Germany) was used, and the values were recorded to the nearest 0.1 cm. All parameters were obtained in routine practice by trained and experienced clinic staff using a standardized protocol. Equipment was calibrated in accordance with hospital quality control procedures.

#### 2.3.4 Laboratory Assays

Fasting venous blood samples (following a 10‐h fast) were centrifuged within 2 h after collection. Plasma glucose levels were measured using the hexokinase/glucose‐6‐phosphate dehydrogenase method (Cobas 8000, Roche Diagnostics), with a coefficient of variation (CV) of less than 2%. Total cholesterol, triglycerides, high‐density lipoprotein cholesterol (HDL‐C), and low‐density lipoprotein cholesterol (LDL‐C) were assessed enzymatically using a Roche Modular P analyzer (CV < 2.5%). All tests were assayed in the laboratory of the Department of Clinical Pathology, the ISO 15189‐ and ISO 15190‐certified laboratories.

### 2.4 Outcome

#### 2.4.1 Primary Outcome

Diagnostic performance of ABSI, AVI, BAI, BMI, BRI, WHR, and WHtR in predicting MetS among Thai women with PCOS.

#### 2.4.2 Secondary Outcome

Performance of composite anthropometric indices for predicting MetS; comparative performance of ABSI, AVI, BAI, BRI, WHR, and WHtR across PCOS phenotypes; and correlations among anthropometric indices and their associations with MetS status.

### 2.5 Statistical Analysis

All statistical analyses were performed using SPSS version 30.0 (SPSS Inc., Chicago, IL, USA). The normality of data distribution was assessed using the Kolmogorov–Smirnov test. Continuous variables are presented as mean ± standard deviation (SD) or median with interquartile range (IQR), as appropriate, and compared using the Student's *t*‐test or the Mann–Whitney *U*‐test. Categorical variables are expressed as frequency (percentage) and compared using the chi‐square test (*χ* ^2^).

The diagnostic performance of each indicator was assessed using the area under the receiver operating characteristic (ROC) curve, the area under the curve (AUC) and Youden's *J* statistic to determine the optimal cut‐off values. ROC curve analysis was conducted with MedCalc software (version 18.2, MedCalc Software Ltd., Ostend, Belgium). Sensitivity, specificity, accuracy, positive predictive value (PPV), negative predictive value (NPV), and odds ratios (OR) were calculated based on the identified cut‐off points. Comparisons of AUCs were performed using DeLong's test. A two‐sided *p*‐value of < 0.05 was considered statistically significant for all analyses. Pearson correlation coefficients were calculated to assess correlations among anthropometric indices and to examine their associations with MetS status.

## 3 Results

**FIGURE 1:**
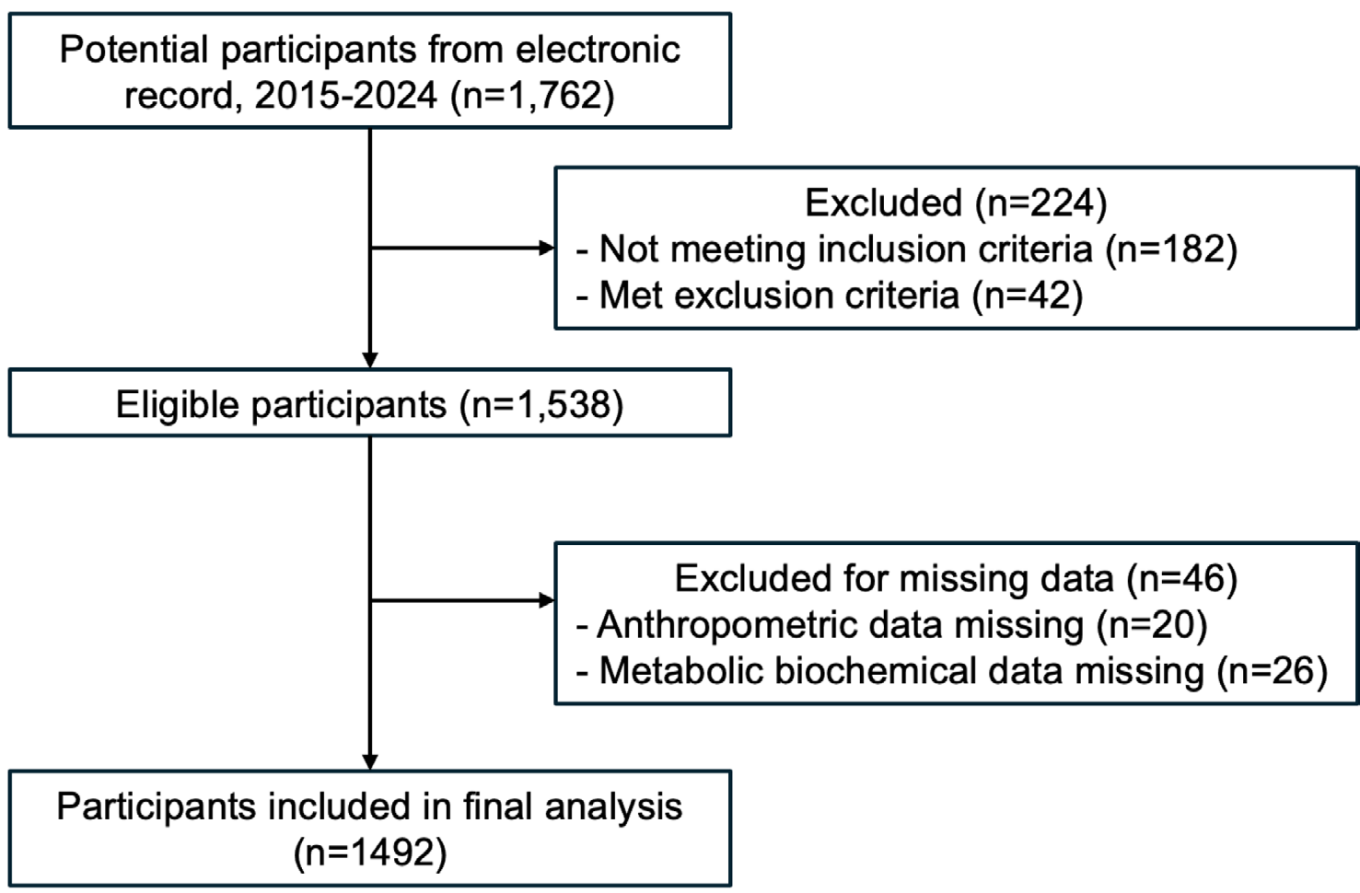
Study flow chart of case selection from the Siriraj PCOS registry and the final analytic sample.

A total of 1538 electronic medical records met all eligibility criteria. Forty‐six cases were excluded due to incomplete data on anthropometric or metabolic biochemical measurements, resulting in 1492 cases included in the final analysis (Figure 1). The baseline clinical and laboratory characteristics, as well as the anthropometric parameters of the study population, were presented in Table 2. The study population had a median [IQR] age of 25 [25, 26, 27, 28, 29] years, and BMI of 25.34 [20.59–31.44] kg/m^2^. Regarding nutritional status, in the overall cohort, 148 (9.9%) were overweight (BMI 23–24.9 kg/m^2^), 298 (20.0%) were obese I (BMI 25–29.9 kg/m^2^), and 474 (31.8%) were obese II (BMI ≥ 30 kg/m^2^). When stratified by metabolic syndrome status, the MetS group had a markedly higher proportion of obese II participants compared with the No‐MetS group (221/311, 71.3% vs 253/1,181, 21.4%), whereas overweight status was less common in the MetS group (15/311, 4.8% vs 133/1,181, 11.3%), and obese I accounted for 74/311 (23.3%) in MetS versus 224/1,181 (19.0%) in No‐MetS.

**TABLE 2:** Baseline characteristics of all PCOS cases with and without MetS.

Variables	Median (IQR)			*p* ^a^
	MetS, *N* = 311	No‐MetS, *N* = 1181	All, *N* = 1492	
Age (years)	27.00 (25–31)	24.00 (21–28)	25 (25–29)	< 0.001
SBP (mmHg)	132 (122–140)	116 (108–125)	119.00 (110–130)	< 0.001
DBP (mmHg)	84 (74–90)	71 (64–78)	73 (66–81)	< 0.001
Body weight (kg)	85.00 (73.90–98.00)	58.90 (50.90–72.95)	64.90 (52.40–81.00)	< 0.001
Height (cm)	160 (157–165)	160 (156–164)	160 (156–164)	
WC (cm)	99 (91–108)	79 (70.5–91.0)	84 (73–96)	< 0.001
HC (cm)	111 (104–120)	96 (89–106)	100 (90–110)	< 0.001
ABSI (m^11/6^/kg^2/3^)	0.077 (0.074–0.079)	0.076 (0.073–0.079)	0.076 (0.073–0.079)	< 0.001
AVI (cm^2^)	19.65 (16.69–23.80)	12.60 (10.17–16.64)	14.20 (10.84–18.47)	< 0.001
BAI	36.41 (32.97–40.08)	29.55 (26.21–34.37)	31.26 (27.16–36.26)	< 0.001
BMI (kg/m^2^)	32.49 (29.26–36.93)	23.32 (20.00–28.56)	25.34 (20.59–31.44)	< 0.001
BRI	5.73 (4.81–7.13)	3.24 (2.39–4.69)	3.83 (2.57–5.42)	< 0.001
WHR	0.89 (0.86–0.94)	0.82 (0.78–0.87)	0.84 (0.79–0.89)	< 0.001
WHtR	0.61 (0.57–0.67)	0.49 (0.45–0.57)	0.52 (0.46–0.60)	< 0.001
Fasting plasma glucose (mg/dL)	95 (86–106)	82 (78–88)	84 (79–91)	< 0.001
Total cholesterol (mmol/L)	197 (175–224)	189 (169–189)	191 (170–213)	< 0.001
Triglyceride (mmol/L)	179 (179–223)	80 (57–110)	91 (62–139)	< 0.001
HDL‐C (mmol/L)	41 (36–46)	62 (52–75)	57 (45–71)	< 0.001
LDL‐C (mmol/L)	119 (97–145)	107 (89–128)	110 (90–131)	< 0.001

Abbreviations: ABSI, a body shape index; AVI, abdominal volume index; BAI, body adiposity index; BMI, body mass index; BRI, body roundness index; DBP, diastolic blood pressure; HC, hip circumference; HDL‐C, high‐density lipoprotein cholesterol; IQR, interquartile range; LDL‐C, low‐density lipoprotein cholesterol; MetS, metabolic syndrome; PCOS, polycystic ovary syndrome; SBP, systolic blood pressure; WC, waist circumference; WHR, waist‐to‐hip; WHtR, waist‐to‐height ratio.

^a^ Comparison of groups with and without metabolic syndrome.

The overall prevalence of MetS using the IDF 2006 definition in this PCOS population was 20.8% (311/1492). The distribution of PCOS phenotypes in the total cohort was as follows: phenotype A (ovulatory dysfunction [OD] + hyperandrogenism/hyperandrogenemia [HA] + polycystic ovarian morphology [PCOM]) in 571 women (38.3%), phenotype B (OD + HA) in 456 (30.6%), phenotype C (OD + PCOM) in 412 (27.6%), and phenotype D (HA + PCOM) in 53 (3.6%).

For all anthropometric indices (Table 2), median values were consistently higher in the MetS group (all *p* < 0.001), including AVI (19.65 [16.69–23.80] vs. 12.60 [10.17–16.64]), BAI (36.41% [32.97–40.08] vs. 29.55% [26.21–34.37]), BMI (32.49 [29.26–36.93] vs. 23.32 [20.00–28.56]), BRI (5.73 [4.81–7.13] vs. 3.24 [2.39–4.69]), WHR (0.89 [0.86–0.94] vs. 0.82 [0.78–0.87]), and WHtR (0.61 [0.57–0.67] vs. 0.49 [0.45–0.57]). ABSI differed only minimally (0.077 [0.074–0.079] vs. 0.076 [0.073–0.079]).

**FIGURE 2:**
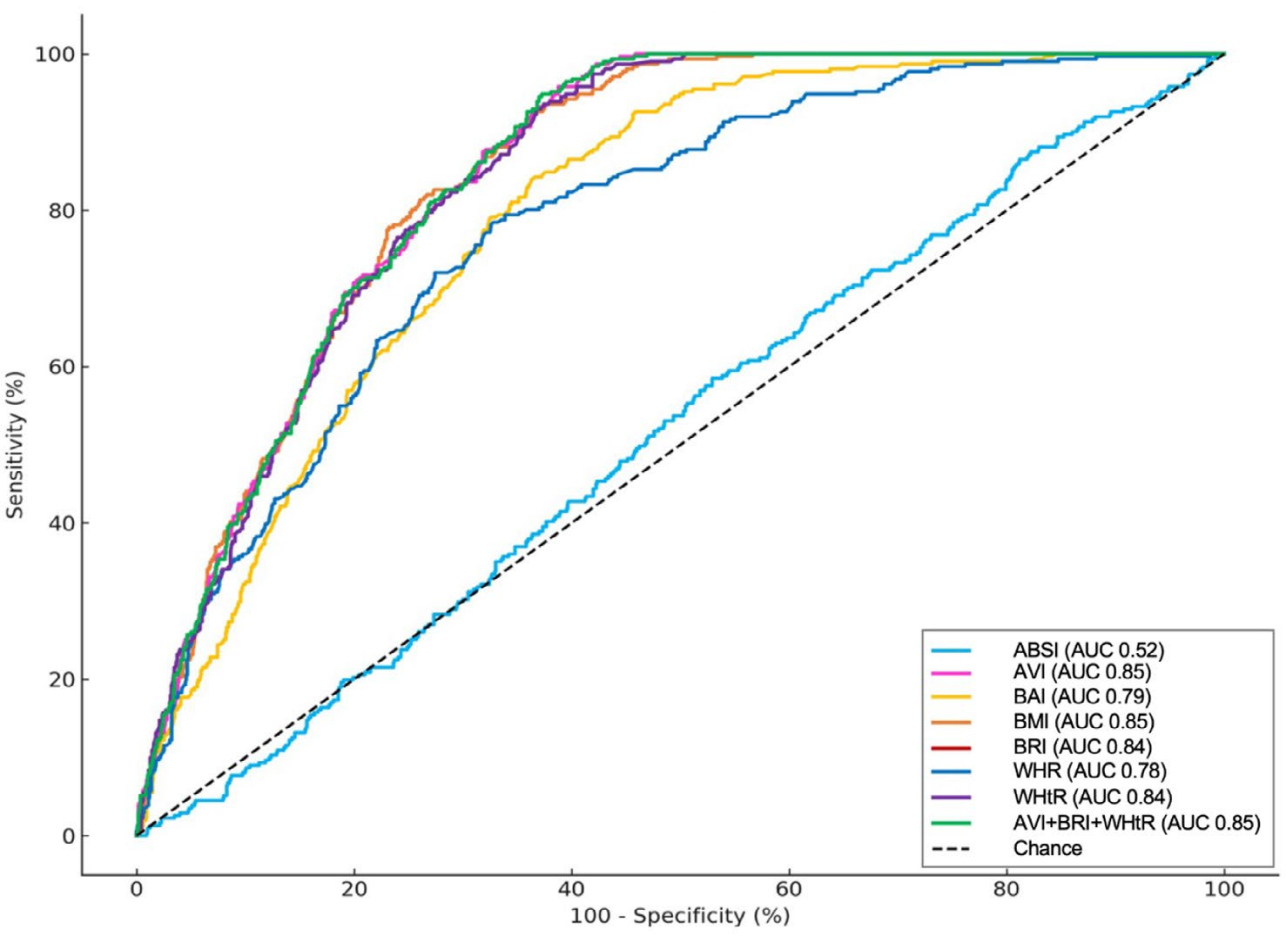
Receiver operating characteristic curves illustrating the discriminative performance of anthropometric indices. A body shape index (ABSI), abdominal volume index (AVI), body adiposity index (BAI), body mass index (BMI), body roundness index (BRI), waist‐to‐hip ratio (WHR), waist‐to‐height ratio (WHtR), and the combined index (AVI + BRI + WHtR) for identifying metabolic syndrome among Thai women with polycystic ovary syndrome. The diagonal dashed line denotes chance performance. Curves closer to the upper‐left corner indicate better discrimination; corresponding area under the curve (AUC) values are presented in Table 3.

ROC analyzes showed that AVI, BMI, BRI, and WHtR provided good discrimination for MetS (AUCs 0.84–0.85), while BAI and WHR were modest (AUCs 0.78–0.79) and ABSI performed poorly (AUC 0.52) (Table 3 and Figure 2). At Youden optimized thresholds, AVI > 14.1 cm^2^, BMI > 25.78 kg/m^2^, BRI > 3.89 and WHtR > 0.53 all achieved sensitivities ≥ 92% with specificities around 63%. NPV exceeded 97% for these four indices, whereas PPV clustered near 40% (reflecting the cohort's 21% MetS prevalence). A simple composite of indices with high AUCs (AVI + BRI + WHtR) was selected to evaluate whether combining measures would enhance discriminative performance. However, this approach did not improve discrimination beyond that of the best‐performing single indices (AUC 0.85; cut‐off > 18.71; sensitivity 94.0%, specificity 62.7%, PPV 37.8%, NPV 93.8%, accuracy 67.9%; OR 3.55) (Table S1). AVI, BRI, and WHtR also demonstrated the strongest discriminative ability across all PCOS phenotypes (Table S2).

**TABLE 3:** Diagnostic performance of anthropometric measurements and indices for the prediction of metabolic syndrome in women with polycystic ovary syndrome.

Index	AUC (95% CI)	*p*	Cut‐off value	Sensitivity (%), (95% CI)	Specificity (%), (95% CI)
ABSI	0.52 (0.48–0.55)	0.523	> 0.076	58.5 (51.9–63.1)	47.1 (44.4–50.1)
AVI	0.85 (0.83–0.87)	< 0.001	> 14.10	95.8 (93.0–97.8)	61.2 (58.4–64.0)
BAI	0.79 (0.77–0.82)	< 0.001	> 31.92	83.9 (79.4–87.8)	63.7 (60.9–66.4)
BMI	0.85 (0.83–0.87)	< 0.001	> 25.78	92.6 (89.1–95.3)	63.6 (60.8–66.3)
BRI	0.84 (0.82–0.86)	< 0.001	> 3.88	93.6 (90.2–96.0)	62.6 (59.7–65.3)
WHR	0.78 (0.75–0.80)	< 0.001	> 0.85	78.5 (74.5–83.8)	67.2 (62.5–68.0)
WHtR	0.84 (0.82–0.86)	< 0.001	> 0.53	93.6 (89.1–95.3)	62.6 (60.4–66.0)
AVI + BRI + WHtR	0.85 (0.83–0.87)	< 0.001	> 18.71	94.0 (91.8–97.0)	62.7 (59.8–65.4)

Abbreviations: ABSI, a body shape index; AUC, area under the curve; AVI, abdominal volume index; BAI, body adiposity index; BMI, body mass index; BRI, body roundness index; CI, confidence interval; WHR, waist‐to‐hip; WHtR, waist‐to‐height ratio.

Pearson correlations (Table 4) demonstrated very strong relationships among waist‐derived indices (e.g., WHtR with BRI; *r* 0.99) and strong correlations between BMI and other waist‐related measures. The correlations between individual indices and MetS status were in the moderate range (approximately *r* 0.39–0.49) for most indices, whereas ABSI showed a near‐null correlation with MetS (*r* 0.02).

**TABLE 4:** Pearson correlation matrix of anthropometric indices.

(*r*)	ABSI	AVI	BAI	BMI	BRI	WHR	WHtR	MetS
ABSI	1.000	0.188	−0.056	−0.152	0.197	0.538	0.207	0.017
AVI	0.188	1.000	0.846	0.927	0.976	0.693	0.971	0.488
BAI	−0.056	0.846	1.000	0.903	0.890	0.373	0.895	0.400
BMI	−0.152	0.927	0.903	1.000	0.925	0.537	0.926	0.487
BRI	0.197	0.976	0.890	0.925	1.000	0.719	0.994	0.482
WHR	0.538	0.693	0.373	0.537	0.719	1.000	0.734	0.392
WHtR	0.207	0.971	0.895	0.926	0.994	0.734	1.000	0.486
MetS	0.017	0.488	0.400	0.487	0.482	0.392	0.486	1.000

Abbreviations: ABSI, a body shape index; AVI, abdominal volume index; BAI, body adiposity index; BMI, body mass index; BRI, body roundness index; MetS, metabolic syndrome; WHR, waist‐to‐hip ratio; WHtR, waist‐to‐height ratio.

## 4 Discussion

In the present cohort of Thai women with PCOS aged 15–45 years, the accuracy of seven anthropometric indices to detect MetS differed significantly. AVI, BMI, BRI, and WHtR demonstrated the strongest discrimination (AUCs 0.84–0.85), with operating thresholds that favor sensitivity for clinical prediction: AVI > 14.10 cm^2^ (AUC 0.85; 95.8%; 61.2%), BMI > 25.78 kg/m^2^ (AUC 0.85; sensitivity 92.6%; specificity 63.6%), BRI > 3.88 (AUC 0.84; 93.6%; 62.6%), and WHtR > 0.53 (AUC 0.84; 93.6%; 62.6%). BAI and WHR were modest (AUCs 0.78–0.79), and ABSI was essentially uninformative (AUC 0.52). Notably, a simple combination (AVI + BRI + WHtR) did not improve AUC beyond those single indices (AUC 0.85), consistent with high collinearity among waist‐based metrics (e.g., WHtR with BRI = 0.99 in our correlation matrix). AVI, BRI, and WHtR were also the strongest discriminations in all phenotypes of PCOS. MetS prevalence in this PCOS cohort was 20.8%, with expected between‐group differences in adiposity and cardiometabolic markers.

This study's rank order aligns with Thai PCOS evidence indicating that central adiposity is the dominant driver of MetS. In a PCOS case–control study in Thailand, Techatraisak et al. reported that BMI, visceral adiposity index (VAI), and WHtR were the strongest predictors of MetS, with optimal cut‐offs of ≥ 28 kg/m^2^ for BMI, > 5.6 for VAI, and ≥ 0.5 for WHtR. BMI demonstrated the highest discriminative performance (AUC 0.90) [31]. The present BMI threshold (25.78 kg/m^2^) is lower, possibly reflecting the use of multiple alternative indices that capture central fat more directly, and our choice of high sensitivity cut points for prediction. Earlier Thai data similarly reported a MetS prevalence of 21% in PCOS by the IDF 2006 criteria, which increased with age and BMI [6]. Global syntheses further confirm that PCOS carries a twofold higher odds ratio of MetS compared with controls, with low HDL and increased WC being the most common components [32, 33].

Physiologically, PCOS combines androgen excess with intrinsic insulin resistance, promoting visceral fat deposition and dyslipidemia. Consequently, indices that capture body roundness (e.g., BRI), approximate abdominal volume (e.g., AVI), or scale WC to body frame (e.g., WHtR) may track risk more closely than weight normalized measures alone [34, 35]. Prior studies have consistently supported WHtR's pragmatic boundary near 0.5 across ethnicities and outcomes, often showing a superior predictive ability compared to BMI for cardiometabolic risk [36, 37, 38]. In our study, however, BMI and WHtR yielded similar outcomes. AVI demonstrated a strong performance (AUC 0.85; sensitivity 95.8%), consistent with its original purpose of reflecting abdominal volume and its association with glycemic risk [39]. Likewise, BRI derived from an ellipsoidal model of WHtR correlated with visceral adiposity and performed comparably to WHtR in our cohort [40].

In contrast, ABSI deliberately downweights WC after adjustment for BMI and height to capture a “body shape” component that is largely orthogonal to overall body size. In PCOS, where the absolute degree of central adiposity is a primary driver of MetS risk, this mathematical construction may suppress clinically relevant information and thereby weaken predictive performance. Accordingly, ABSI should be interpreted with caution in similar populations. Consistent with our null result for ABSI (AUC 0.52), a prior study in PCOS likewise found that WHtR predicted MetS well, whereas ABSI did not [41]. In addition, reviews in the general population have frequently reported that ABSI underperforms simple waist‐based indices for predicting MetS and its components [42].

Our thresholds of BMI ≥ 25.78 kg/m^2^ and WHtR ≥ 0.53 offer pragmatic cut‐points for first‐line triage in Thai PCOS clinics and are feasible in resource‐limited settings. WHtR is especially attractive because it can be self‐assessed using only a tape measure and height, supporting a simple public message of keeping WC below approximately half of height, and facilitating community counseling and lifestyle programs. These thresholds are also consistent with the IDF emphasis on central obesity (WC ≥ 80 cm for Asian women) [19].

In our data, BMI and WHtR provided workflow advantages with high sensitivity (92.6%–93.6%) and excellent NPV (97.0%–97.9%), supporting their use as first‐line screening tools for MetS prediction. AVI achieved the highest sensitivity (95.8%) but requires precise circumference measurement and calculation, making it a reasonable second‐line option where anthropometric quality is assured. BRI performed similarly to WHtR and may be used interchangeably when automated calculators are available [40]. Given the near equivalent predictive value of waist‐based indices, combining measures adds only little value to AUC while needing much more complicating workflows. Thus, selecting a single simple index is the most efficient approach.

A streamlined pathway is proposed for Thai PCOS clinics and other settings with limited access to laboratory testing. The first step, triage the patient using BMI or WHtR at the proposed cut‐offs. The second step, if the first step is positive, evaluate IDF MetS components (blood pressure, fasting plasma glucose, triglycerides, and HDL‐C) and counsel aggressively on lifestyle and weight management. The third step, prioritize central obesity and older age within PCOS phenotypes when allocating preventive resources [33]. This approach harmonizes with the IDF's central obesity anchored definition and the 2023 international PCOS guideline that emphasizes the assessment of cardiometabolic risk [19, 27].

Although our primary outcome was MetS, these anthropometric thresholds may also support counseling in reproductive settings by identifying women who may benefit from earlier metabolic optimization before conception and assisted reproduction. Early recognition of MetS in reproductive‐aged women is particularly important because clustering of metabolic risk factors before or during pregnancy is associated with adverse maternal outcomes, including gestational diabetes mellitus and preeclampsia [43]. Thus, beyond the PCOS context, scalable strategies for earlier detection of MetS in reproductive‐aged women may help reduce long‐term cardiometabolic burden while improving reproductive and pregnancy‐related outcomes.

Excess body weight not only worsens cardiometabolic risk but may also impair reproductive potential through interactions among adiposity, hormonal profiles, and ovarian function. Vale‐Fernandes et al. reported that PCOS and excessive body weight influence fertility treatment outcomes both independently and synergistically, suggesting that obesity may exacerbate hyperandrogenism and further disrupt fertility in women with PCOS [44]. Assisted reproductive technology studies similarly emphasize that reproductive prognosis in PCOS is multifactorial and that anthropometric measures should be interpreted alongside hormonal and age‐related parameters. In women with PCOS undergoing in vitro fertilization, Ribeiro et al. evaluated anti‐Müllerian hormone (AMH), BMI, age, and the luteinizing hormone: follicle‐stimulating hormone ratio in relation to reproductive outcomes and concluded that AMH should not be used as a sole predictor. Notably, age had a stronger association with live birth than BMI, and cumulative live birth rate was highlighted as the most informative metric for counseling couples [45]. In women with PCOS undergoing intrauterine insemination, Moreira et al. observed a trend toward poorer reproductive outcomes with increasing BMI and suggested BMI may be a useful predictor, while also noting that both low and high AMH may indicate a less favorable prognosis in this setting [46]. Together, these findings support the potential value of integrating simple anthropometric screening with hormonal and reproductive parameters (e.g., AMH and age) to enable more comprehensive risk stratification and patient‐centered counseling in future prospective studies.

Strengths of the present study include the head‐to‐head comparison of seven anthropometric indices in a sizable, single‐ethnicity cohort of a well‐designed registry (the Siriraj PCOS project), applying uniform case definitions for PCOS (Revised Rotterdam 2003) and MetS (IDF 2006). Laboratory assays were performed in a single center, minimizing variation and potential measurement error. We reduced diagnostic bias by excluding potential confounders that could influence MetS ascertainment. Limitations of the present study include its single‐center design, which limits causal inference and may reduce generalizability. Residual confounding from unmeasured lifestyle, dietary, socioeconomic, and physical activity factors remains possible. We conducted a complete‐case analysis without imputation. Although the proportion of missing data was low (3.0%), selection bias cannot be excluded.

Future work should further link these anthropometric indices to hard outcomes (e.g., incident T2DM and CVD) to establish prognostic relevance. Prospective multicenter or multinational Asian studies could validate the cut‐offs, assess longitudinal prediction of cardiovascular outcomes, and explore implementation in mobile health applications. In addition, when developing multivariable risk prediction models that integrate anthropometric, hormonal, and reproductive parameters, studies should explicitly assess model calibration (e.g., agreement between predicted and observed risks) alongside discrimination to ensure clinical reliability across settings and subgroups. Finally, randomized trials are needed to determine whether using the composite index to guide interventions improves metabolic outcomes.

In conclusion, simple waist‐centric indices (AVI, BRI, and WHtR), as well as BMI, provide clinically meaningful discrimination for predicting MetS in Thai women with PCOS, whereas ABSI failed to contribute to discrimination. A single pragmatic screening measure, preferably BMI ≥ 25.78 kg/m^2^ or WHtR ≥ 0.53, appears sufficient for first‐line triage, with confirmatory metabolic evaluation recommended for individuals who screen positive in accordance with current IDF‐based practice.

## Author Contributions

**Marisa Yokyongsakul:** conceptualization, methodology, software, validation, formal analysis, investigation, resources, data curation, writing – original draft, writing – review and editing. **Pavarit Humart:** conceptualization, methodology, software, validation, formal analysis, investigation, resources, data curation, writing – original draft, writing – review and editing, visualization, supervision, project administration. **Kitirat Techatraisak:** conceptualization, methodology, resources, writing – original draft, writing – review and editing, supervision. **Prasong Tanmahasamut:** resources, writing – review and editing. **Manee Rattanachaiyanont:** resources, writing – review and editing. **Suchada Indhavivadhana:** resources, writing – review and editing. **Thanyarat Wongwananuruk:** resources, writing – review and editing. **Panicha Chantrapanichkul:** resources, writing – review and editing. **Matus Phunyammalee:** resources, writing – review and editing. **Pornpimol Madeesukstit:** data curation. All authors have read and agreed to the published version of the manuscript.

## Funding

This study was supported by funding for Institutional Review Board submission from the Faculty of Medicine Siriraj Hospital, Mahidol University.

## Ethics Statement

The study was conducted in accordance with the Declaration of Helsinki and approved by the Siriraj Hospital Institutional Review Board (COA no. Si 927/2024).

## Consent

The requirement for written informed consent was waived by the Institutional Review Board due to the retrospective design.

## Conflicts of Interest

The authors declare no conflicts of interest.

## Supporting information


**Table S1:** Diagnostic performance of anthropometric measurements and indices for the prediction of metabolic syndrome in women with polycystic ovary syndrome.
**Table S2:** Correlation between anthropometric measures and PCOS phenotypes in detecting metabolic syndrome.

## Data Availability

The data supporting the findings of this study are derived from a hospital‐based retrospective registry and contain potentially identifiable clinical information. Due to ethical restrictions imposed by the Siriraj Institutional Review Board and national data protection regulations, the data cannot be made publicly available or deposited in a public repository. De‐identified data may be made available for scientific purposes upon reasonable request and subject to approval by the Siriraj Institutional Review Board and the corresponding author.
