# Supplementary material for: Anthropometric Measurements to Predict Metabolic Syndrome in Thai Women With Polycystic Ovary Syndrome: A Retrospective Study
Source: J Obstet Gynaecol Res. 2026 Mar 19;52(3):e70255. doi: 10.1111/jog.70255 (PMC13002327; doi:10.1111/jog.70255)
Supplement: Supplementary file 1 — Table S1: Diagnostic performance of anthropometric measurements and indices for the prediction of metabolic syndrome in women with polycystic ovary syndrome. Table S2: Correlation between anthropometric measures and PCOS phenotypes in detecting metabolic syndrome. [file JOG-52-0-s001.docx]

| **TABLE S1** \| Diagnostic performance of anthropometric measurements and indices for the prediction of metabolic syndrome in women with polycystic ovary syndrome | | | | | |
| --- | --- | --- | --- | --- | --- |
| **Index** | **Cut-off value** | **PPV, %** | **NPV, %** | **Accuracy, %** | **OR per 1 SD** |
| ABSI | >0.076 | 22.6 | 81.2 | 49.5 | 1.04 |
| AVI | >14.10 | 39.5 | 98.2 | 68.4 | 3.52 |
| BAI | >31.92 | 37.8 | 93.8 | 67.9 | 2.83 |
| BMI | >25.78 | 40.1 | 97.0 | 69.6 | 3.69 |
| BRI | >3.88 | 39.7 | 97.4 | 69.0 | 3.50 |
| WHR | >0.85 | 37.3 | 92.3 | 68.0 | 2.99 |
| WHtR | >0.53 | 40.0 | 97.9 | 69.4 | 3.92 |
| AVI + BRI + WHtR | >18.71 | 37.8 | 93.8 | 67.9 | 3.55 |
| Abbreviations: PPV, positive predictive value; NPV, negative predictive value; OR, odds ratio; SD, standard deviation; BAI, body adiposity index; BMI, body mass index; BRI, body roundness index; AVI, abdominal volume index; ABSI, a body shape index; WHR, waist-to-hip; WHtR, waist-to-height ratio | | | | | |

| **TABLE S2** \| Correlation between anthropometric measures and PCOS phenotypes in detecting metabolic syndrome | | | | | |
| --- | --- | --- | --- | --- | --- |
| **Index** | **AUC** | ***p* value** | **Cut off value** | **Sensitivity, %** | **Specificity, %** |
| **Phenotype A (N 123/571)** | | | | | |
| ABSI | 0.53 | 0.296 | >0.080 | 82.9 | 24.8 |
| AVI | 0.84 | <0.001 | >13.33 | 99.2 | 59.4 |
| BAI | 0.78 | <0.001 | >29.45 | 94.3 | 52.7 |
| BRI | 0.83 | <0.001 | >3.56 | 96.7 | 61.4 |
| WHR | 0.75 | <0.001 | >0.86 | 68.3 | 71.0 |
| WHtR | 0.83 | <0.001 | >0.51 | 96.7 | 61.4 |
| **Phenotype B (N 88/368)** | | | | | |
| ABSI | 0.46 | 0.200 | >0.083 | 96.6 | 6.0 |
| AVI | 0.84 | <0.001 | >15.15 | 90.9 | 67.7 |
| BAI | 0.78 | <0.001 | >32.93 | 84.1 | 65.8 |
| BRI | 0.84 | <0.001 | >3.93 | 97.7 | 62.0 |
| WHR | 0.80 | <0.001 | >0.85 | 81.8 | 70.9 |
| WHtR | 0.84 | <0.001 | >0.53 | 97.0 | 62.0 |
| **Phenotype C (N 95/317)** | | | | | |
| ABSI | 0.45 | 0.104 | >0.082 | 91.6 | 13.6 |
| AVI | 0.85 | <0.001 | >14.24 | 96.8 | 60.3 |
| BAI | 0.81 | <0.001 | >31.92 | 88.4 | 64.0 |
| BRI | 0.85 | <0.001 | >4.93 | 80.0 | 75.7 |
| WHR | 0.79 | <0.001 | >0.85 | 81.1 | 65.9 |
| WHtR | 0.85 | <0.001 | >0.58 | 80.0 | 75.7 |
| **Phenotype D (N 5/48)** | | | | | |
| ABSI | 0.44 | 0.622 | >0.080 | 100.0 | 22.9 |
| AVI | 0.94 | <0.001 | >18.26 | 100.0 | 89.6 |
| BAI | 0.89 | <0.001 | >32.79 | 100.0 | 75.0 |
| BRI | 0.94 | <0.001 | >5.22 | 100.0 | 17.0 |
| WHR | 0.87 | <0.001 | >0.89 | 80.0 | 17.0 |
| WHtR | 0.94 | <0.001 | >0.59 | 100.0 | 17.0 |
| Abbreviations: AUC,area under the curve; SD, standard deviation; PPV,positive predictive value; NPV,negative predictive value; OR,odds ratio; BAI,body adiposity index; BMI,body mass index; BRI,body roundness index; AVI,Abdominal volume index; ABSI,a body shape index; WHR,waist to hip; WHtR,waist to height ratio | | | | | |
